# Prophylactic dexamethasone for the prevention of radiation-induced pain flare in patients with bone metastases: a meta-analysis

**DOI:** 10.3389/fonc.2026.1894776

**Published:** 2026-08-14

**Authors:** Lang Chen, YaJun Wang, Jian Tan, MingLiu He, Han Gou, Xing Chen, YiHui Liu

**Affiliations:** Dazhou Dachuan District People’s Hospital, (Dazhou Third People’s Hospital), Dazhou, China

**Keywords:** bone metastases, dexamethasone, meta-analysis, pain flare, radiotherapy

## Abstract

**Purpose:**

This study primarily evaluated the efficacy and safety of prophylactic dexamethasone in preventing radiation-induced pain flare in patients with bone metastases undergoing palliative radiotherapy.

**Methods:**

A systematic search of the PubMed, Embase, and Cochrane databases was conducted to identify prospective randomized controlled trials published up to May 2026. After screening, six studies were ultimately included. A random-effects model was used for pooled analyses, and sensitivity analyses were performed to assess heterogeneity.

**Results:**

A total of 1,833 records were initially identified, and six prospective randomized controlled trials were ultimately included in the final analysis. Pooled analysis demonstrated that prophylactic dexamethasone was associated with a significantly lower risk of radiation-induced pain flare compared with placebo (RR = 0.63, 95% CI: 0.46–0.86). Sensitivity analyses did not identify a clear source of heterogeneity within the dexamethasone group. Adverse events were infrequently reported and were generally mild, with no severe treatment-related complications observed in the included studies.

**Conclusion:**

Prophylactic dexamethasone may reduce the incidence of radiation-induced pain flare in patients with bone metastases undergoing palliative radiotherapy. However, given the limited number of studies and heterogeneity among treatment protocols, these findings should be interpreted cautiously, and further large-scale randomized controlled trials are warranted.

## Introduction

Cancer remains one of the leading causes of death worldwide. Malignant tumor cells can detach from the primary lesion and spread to distant organs through a complex metastatic process, among which the skeleton is one of the most common sites of metastasis (1, 2). Bone metastases are frequently associated with severe complications, including refractory bone pain, pathological fractures, spinal cord compression, and hypercalcemia, all of which can significantly impair patients’ quality of life and negatively affect overall survival (3). At present, radiotherapy (RT) is considered one of the most effective palliative treatment modalities for patients with bone metastases. RT can effectively relieve cancer-related bone pain, reduce local tumor burden, and delay the occurrence of skeletal-related events, thereby improving functional status and quality of life (4). However, despite its clinical benefits, a proportion of patients experience a transient exacerbation of pain shortly after radiotherapy, commonly referred to as “pain flare” (5). This phenomenon not only aggravates patients’ physical discomfort and psychological burden, but may also negatively affect treatment adherence and the overall therapeutic experience (6). Therefore, the prevention and management of radiation-induced pain flare has become an important clinical concern in the supportive care of patients with bone metastases.

Dexamethasone has been widely used as an adjuvant analgesic agent in the management of cancer-related pain because of its potent anti-inflammatory and anti-edema effects (7–9). Previous studies investigating the role of dexamethasone in preventing radiation-induced pain flare have reported inconsistent findings. Hird et al. observed that prophylactic dexamethasone administration was associated with a lower incidence of pain flare after palliative radiotherapy for bone metastases, indicating a possible benefit of corticosteroid intervention in symptom control and treatment tolerance (10). In contrast, Tonev et al. did not identify a significant preventive effect of dexamethasone in routine clinical practice (11). The discrepancy among studies may be attributable to variations in study design, radiotherapy protocols, dexamethasone regimens, patient characteristics, and outcome evaluation methods. Consequently, the clinical value of dexamethasone in preventing radiation-induced pain flare remains uncertain.

Therefore, the present meta-analysis was conducted to systematically assess the efficacy and safety of prophylactic dexamethasone compared with placebo in patients with bone metastases receiving palliative radiotherapy. It is anticipated that the findings of this study may contribute additional evidence for clinical decision-making and provide a reference for the rational application of corticosteroids in supportive cancer care.

## Material and methods

### Search strategy

Relevant studies published up to May 2026 were systematically identified through searches of the PubMed, Embase, and Cochrane databases. The search strategy combined terms related to “radiotherapy,” “bone metastases,” and “dexamethasone.”

### Inclusion and exclusion criteria

The following inclusion criteria were applied:

1. Randomized controlled trials (RCTs) reporting original data.
2. Studies meeting the following Participants, Intervention, Comparator, and Outcome (PICO) criteria:
  a. Participants (P): adult patients (≥18 years) with bone metastases secondary to malignant tumors who received palliative radiotherapy.
  b. Intervention (I): prophylactic dexamethasone or other glucocorticoid administration.
  c. Comparator (C): placebo, saline infusion, or no prophylactic treatment.
  d. Outcome (O): incidence of radiation-induced pain flare following radiotherapy.

The following exclusion criteria were applied:

1. Grey literature, including conference abstracts, non-peer-reviewed documents, preprints, or unpublished studies.
2. Articles without accessible full texts.
3. Studies not published in English.

### Quality assessment

The quality of the included studies was independently assessed by two investigators using the Cochrane Risk of Bias tool. Each study was evaluated for potential risk of bias across relevant methodological domains and categorized as having low, high, or unclear risk of bias. Any disagreements between the reviewers were resolved through discussion with a third investigator. Quality assessment and graphical visualization were performed using RevMan software (version 5.3).

### Data extraction

Data extraction from all included studies was independently performed by two investigators. The extracted data included the following: (1) study information, including the first author and year of publication; (2) study characteristics, including country of origin and study design; (3) patient characteristics, including sample size, mean/median age, and baseline worst pain score; and (4) technical characteristics, including radiotherapy dose and intervention drug dosage. When relevant information was not explicitly reported in the text, data were manually extracted from the tables, figures, and supplementary materials whenever possible.

### Statistical analysis

Statistical analyses were conducted using RStudio software (version 4.3.0). The primary outcome was the incidence of radiation-induced pain flare following palliative radiotherapy. For dichotomous outcomes, treatment effects were summarized as risk ratios (RRs) with corresponding 95% confidence intervals (CIs). Pooled estimates were calculated using a random-effects model to account for potential clinical and methodological heterogeneity among the included studies. Statistical heterogeneity was assessed using the I² statistic, with values greater than 50% considered indicative of substantial heterogeneity. Forest plots were generated to visualize pooled effect estimates and between-study variability. Sensitivity analyses were performed using a leave-one-out approach, in which individual studies were sequentially excluded to evaluate the robustness of the pooled results and to identify potential sources of heterogeneity. Statistical significance was considered present when the 95% confidence interval did not include the null value (RR = 1.0).

## Results

### Study selection

**Figure 1:**
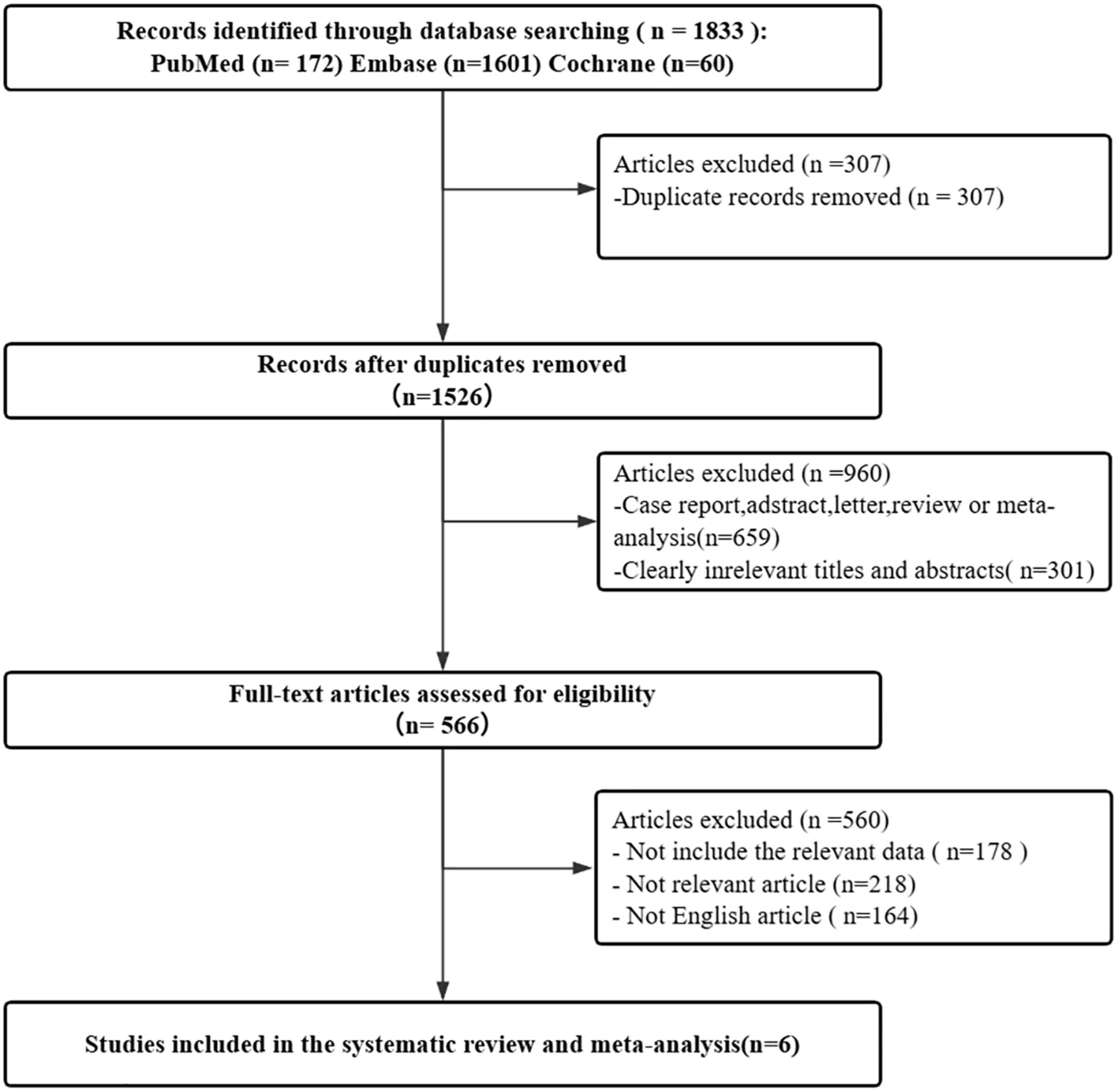
Study selection flow diagram.

A total of 1,833 records were initially identified through the literature search. After removing 307 duplicate articles, 1,526 records remained for screening. Among these, 659 publications, including case reports, conference abstracts, reviews, and meta-analyses, as well as 301 records deemed clearly irrelevant based on titles and abstracts, were excluded. The remaining 566 studies underwent full-text eligibility assessment. Subsequently, 178 studies were excluded due to insufficient extractable data, 218 studies were excluded for not being relevant to the study objective, and 164 studies were excluded because they were not published in English. Ultimately, six studies were included in the final analysis (12–17). The study selection process is illustrated in the flow diagram (**Figure 1**).

### Quality assessment

**Figure 2:**
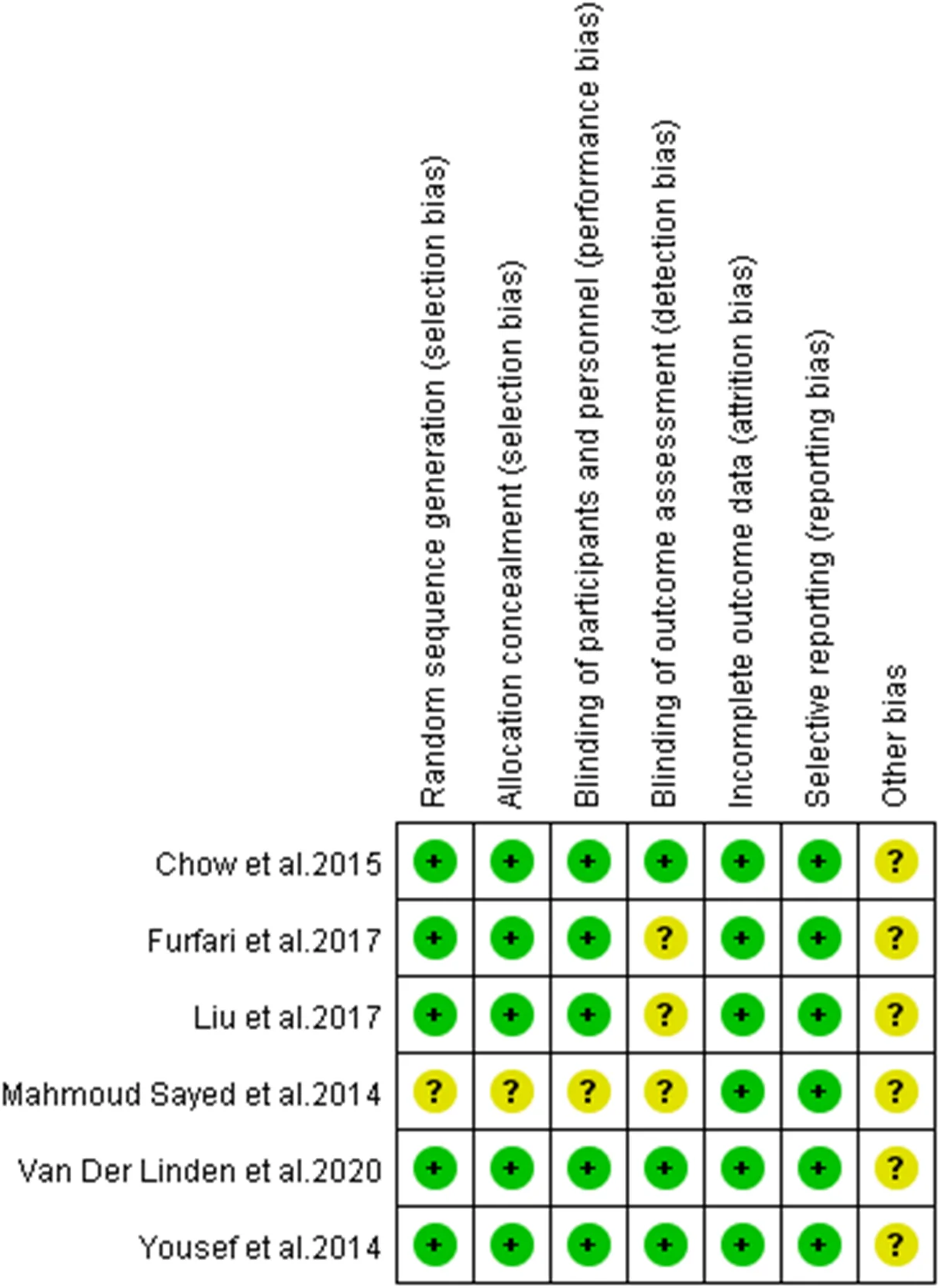
Quality assessment of the included studies using the Cochrane Risk of Bias tool.

The risk of bias of the included studies was assessed using the Cochrane Risk of Bias tool. As shown in **Figure 2**, most studies were judged to have a low risk of bias across the evaluated methodological domains, although some studies raised concerns regarding incomplete outcome data and reporting of methodological details. Overall, the methodological quality of the included studies was considered acceptable, and the risk of bias was deemed sufficiently low to support their inclusion in the present meta-analysis.

### Study, patient, and technical characteristics

Seven prospective studies involving patients with bone metastases receiving palliative radiotherapy were included. The studies were conducted in Canada, the Netherlands, China, and Egypt, with sample sizes ranging from 81 to 298 patients. Dexamethasone was the main prophylactic intervention, while one study used methylprednisolone. Radiotherapy regimens included both single-fraction and multifraction schedules. Primary tumors mainly included breast, prostate, and lung cancers. Reported adverse events were generally mild. Detailed characteristics are summarized in **Tables 1**, **2**.

**Table 1:** Study and patient characteristics of the included studies.

Author	Year	Study characteristics		Patient characteristics		Baseline worst pain score		
		Country	Study design	No. of patients	Age (years)	2-4	5-6	7-10
Chow et al.	2015	Canada	prospective study	298	69 (32–96)	IG:34 CG:31	IG:41 CG:41	IG:73 CG:78
Furfari et al.	2017	Canada	prospective study	81	72 (33–95)	15	21	45
Van Der Linden et al.	2020	The Netherlands	prospective study	295	NA	NA	NA	NA
Liu et al.	2017	China	prospective study	103	69	NA	NA	IG:25 CG:28
Mahmoud Sayed et al.	2014	Egypt	prospective study	147	55 (23–74)	NA	IG:27 CG:36	IG:41 CG:43
Yousef et al.	2014	Egypt	prospective study	120	IG: 65 (45–78) CG: 62 (50–79)	NA	NA	NA

NA, not available; IG, intervention group; CG, control group.

**Table 2:** Technical characteristics of the included studies.

Author	Year	Intervention	Radiotherapy dose	Intervention dose	Primary lesion	Adverse events
Chow et al.	2015	dexamethasone	1×8Gy	8mg,po (days 1-5)	Breast,Prostate,Lung,Other	hyperglycaemic events(3)
Furfari et al.	2017	dexamethasone	1×8Gy	4 mg × 2,po (1 h before RT and days 1–4)	Prostate,Breast,Lung,Other	NA
Van Der Linden et al.	2020	dexamethasone	1×8 Gy or 5-6×20–24 Gy	8 mg,po dexamethasone (days 1), placebo (days 2–4);8 mg,po dexamethasone (days 1–4)	NA	NA
Liu et al.	2017	dexamethasone	20×2Gy	5mg,iv (days 1-10)	Lung	0
Mahmoud Sayed et al.	2014	dexamethasone	5×4Gy	8mg,po (days 1-4)	Breast,Bladder,Lung,Sarcoma,Other	NA
Yousef et al.	2014	Methyl-prednisolone	3×10Gy	5 mg/kg,iv	Lung,Breast,Gastrointestinal,Liver,Prostate,Other	NA

NA, not available; IG, intervention group; CG, control group; po, oral administration; iv, intravenous administration.

### Efficacy of dexamethasone in preventing radiation-induced pain flare

**Figure 3:**
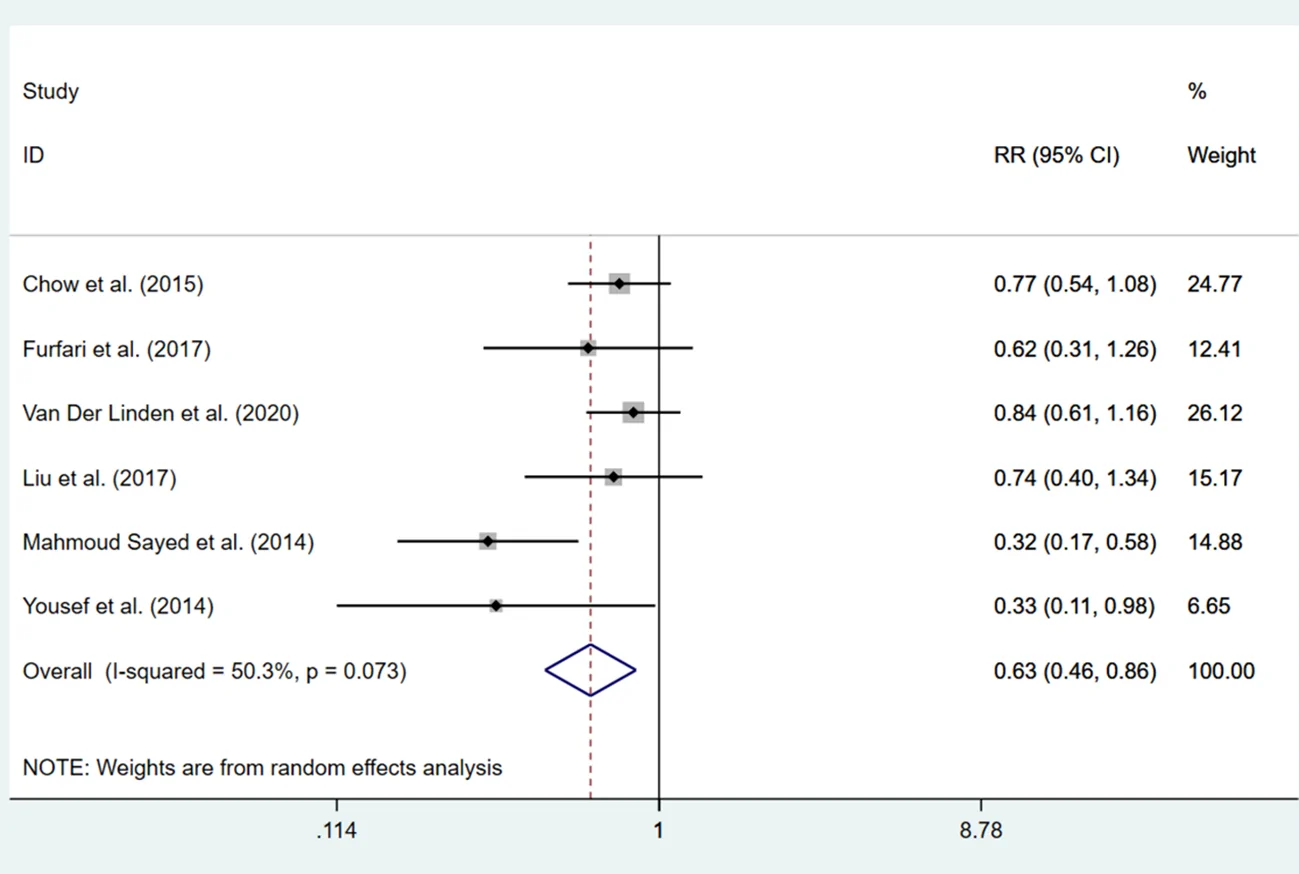
Forest plot comparing dexamethasone versus placebo for the prevention of radiation-induced pain flare following palliative radiotherapy.

The event counts for radiation-induced pain flare in the included studies are summarized in **Table 3**. As shown in **Figure 3**, the pooled analysis demonstrated that prophylactic dexamethasone was associated with a significantly lower risk of radiation-induced pain flare compared with placebo (RR = 0.63, 95% CI: 0.46–0.86). These findings suggest that dexamethasone may be effective in reducing the risk of pain flare following palliative radiotherapy in patients with bone metastases.

**Table 3:** Event counts for radiation-induced pain flare in the included studies.

Study	Year	Eevent	Enoevent	Etotal	Cevent	Cnoevent	Ctotal
Chow et al.	2015	39	109	148	53	101	150
Furfari et al.	2017	11	39	50	11	20	31
Van Der Linden et al.	2020	64	131	195	39	61	100
Liu et al.	2017	13	38	51	18	34	52
Mahmoud Sayed et al.	2014	11	68	79	30	38	68
Yousef et al.	2014	4	56	60	12	48	60

Eevent, number of patients with radiation-induced pain flare in the experimental group; Enoevent, number of patients without radiation-induced pain flare in the experimental group; Etotal, total number of patients in the experimental group; Cevent, number of patients with radiation-induced pain flare in the control group; Cnoevent, number of patients without radiation-induced pain flare in the control group; Ctotal, total number of patients in the control group.

### Heterogeneity analysis

**Figure 4:**
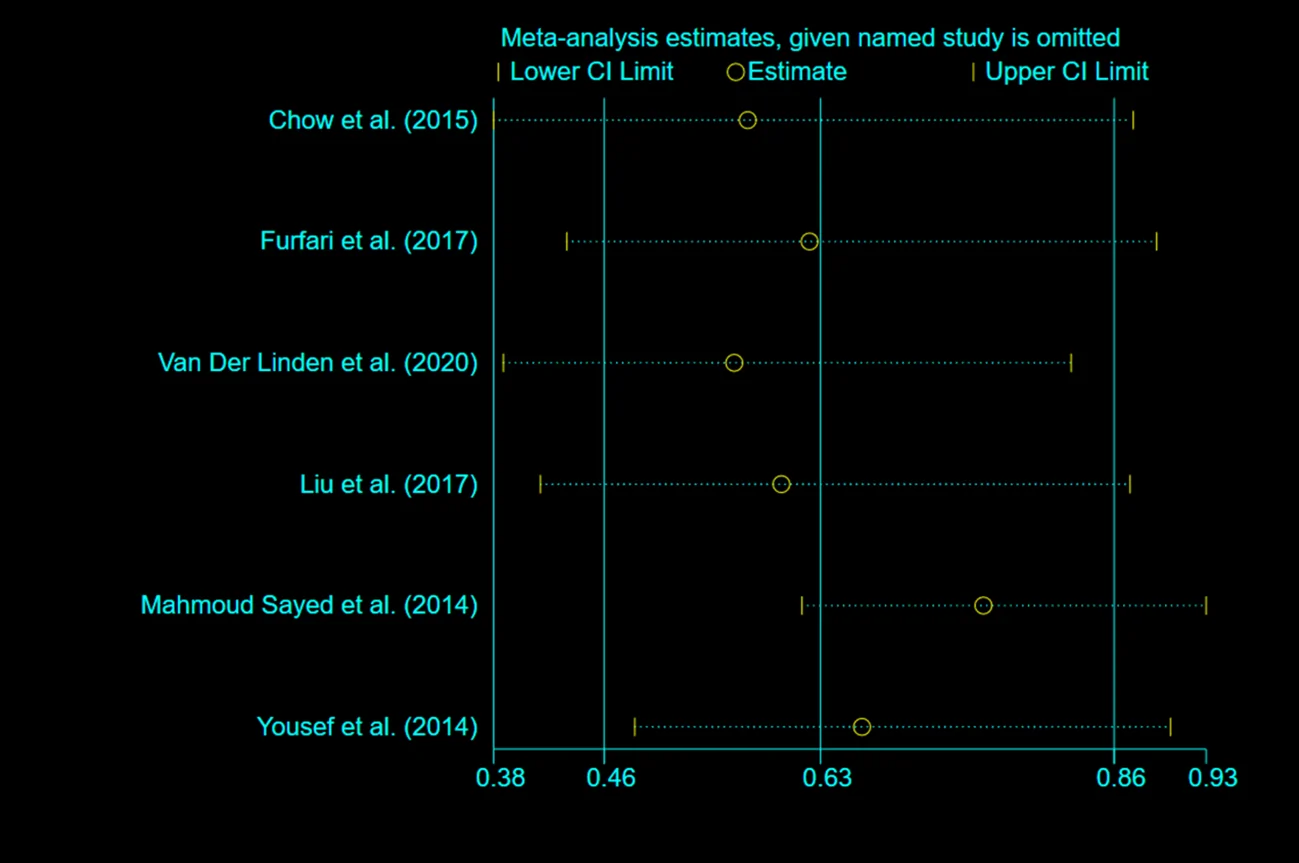
Sensitivity analysis of the pooled risk ratio for radiation-induced pain flare.

As moderate heterogeneity was observed among the included studies, sensitivity analyses were performed to evaluate the robustness of the pooled estimates and to explore potential sources of heterogeneity. As shown in **Figure 4**, sequential omission of individual studies did not result in substantial changes in the overall effect size, indicating that the pooled results were generally stable. Furthermore, no single study was identified as a major contributor to the observed heterogeneity, suggesting that the findings of the present meta-analysis were relatively robust and not unduly influenced by any individual study.

### Adverse events

Adverse events were reported in only a limited number of the included studies. In the study by Chow et al., three cases of hyperglycaemic events were observed among 298 patients receiving dexamethasone prophylaxis, and no severe treatment-related adverse events were reported. In the study conducted by Liu et al., which included 103 patients, no adverse events associated with dexamethasone administration were observed. Overall, the available evidence suggests that prophylactic corticosteroid use may be generally tolerable in patients undergoing palliative radiotherapy for bone metastases. However, given the limited reporting of adverse events across studies, further high-quality research is still needed to more comprehensively evaluate the safety profile of dexamethasone in this setting.

## Discussion

Bone metastases-related pain after radiotherapy can significantly impair patients’ quality of life. Emerging evidence suggests that dexamethasone may help alleviate post-radiotherapy pain and reduce the risk of pain flare (18). In the study by Chow et al., patients receiving single-fraction 8 Gy radiotherapy were given 8 mg dexamethasone prior to treatment, with pain flare assessed using the Brief Pain Inventory over 10 days. The results indicated a potential preventive effect of dexamethasone on radiation-induced pain flare, although further randomized trials were recommended to confirm these findings. Similarly (12), Kalra et al. compared two oral dexamethasone regimens (8 mg vs 4 mg daily) in patients receiving palliative radiotherapy for bone metastases. The 8 mg group showed better pain relief and functional outcomes with reduced analgesic requirements compared with the lower-dose group (19). Overall, dexamethasone appeared to provide clinical benefit with an acceptable safety profile, although further studies are needed to better define its optimal role in this setting.

Radiation-induced pain flare is a relatively common adverse event following radiotherapy in patients with malignant tumors, and it may negatively affect patients’ quality of life and treatment adherence in clinical practice (20–23). The underlying mechanism remains incompletely understood and is considered to be multifactorial. Westhoff et al. suggested that post-radiotherapy pain flare may be associated with inflammatory reactions and edema in irradiated bone and periosteal tissues (6). Increased local tissue pressure and the release of inflammatory mediators may further activate or sensitize nociceptive pathways, thereby contributing to transient exacerbation of pain symptoms. Dexamethasone, a corticosteroid with well-established anti-inflammatory and anti-edematous effects, has been proposed as a potential intervention for reducing radiation-induced pain flare following palliative radiotherapy for bone metastases (24–27). Its potential mechanisms may involve suppression of inflammatory mediator release, reduction of vascular permeability, alleviation of local tissue edema, and modulation of nociceptive sensitization. Nevertheless, the precise biological mechanisms underlying its therapeutic effects remain incompletely understood, and further experimental and clinical investigations are still required.

Regarding safety, the currently available evidence suggests that prophylactic dexamethasone may be generally well tolerated. In the studies conducted by Chow et al. and Liu et al., no severe treatment-related adverse events were reported. Similar observations have also been described in other clinical settings. Zielen et al. reported that dexamethasone-based therapy did not result in serious corticosteroid-related complications or treatment-related mortality (28), while Fan et al. found that most adverse events were mild and manageable (29). Comparable safety findings have also been documented in several other studies (30–32). However, concerns regarding potential adverse effects of dexamethasone under certain clinical conditions have also been raised (33). Therefore, the overall safety profile of prophylactic dexamethasone remains to be further clarified through larger and higher-quality studies.

The present meta-analysis demonstrated that prophylactic dexamethasone was associated with a significantly reduced risk of radiation-induced pain flare compared with placebo in patients with bone metastases undergoing palliative radiotherapy (RR = 0.63, 95% CI: 0.46–0.86). These findings suggest that dexamethasone may provide a clinically meaningful benefit in mitigating post-radiotherapy pain exacerbation and may serve as a useful supportive intervention in this patient population. Nevertheless, the overall certainty of the available evidence remains limited, and the results should therefore be interpreted with caution. Sensitivity analyses demonstrated that the pooled effect estimates were generally stable and robust, although no clear source of heterogeneity could be identified. Therefore, additional well-designed, adequately powered randomized controlled trials are needed to confirm these findings and to inform more definitive clinical recommendations.

Several limitations of the present study should also be acknowledged. First, the number of eligible studies was relatively small, which may have affected the statistical power and robustness of the pooled analysis. Second, there was substantial variability in dexamethasone administration protocols among studies, including differences in dosage, timing, and duration of treatment, potentially contributing to uncertainty in the pooled estimates. Third, subgroup analyses based on clinically relevant factors, such as primary tumor type and radiotherapy regimen, could not be performed because of insufficient data, thereby limiting the generalizability of the findings. In addition, inconsistencies in adverse event reporting and outcome definitions across studies may also have influenced the interpretation of efficacy and safety outcomes. Taken together, these limitations highlight the need for further large-scale, well-designed randomized controlled trials with standardized methodologies to better define the efficacy, safety, and optimal clinical application of dexamethasone in preventing radiation-induced pain flare.

## Conclusion

This meta-analysis suggests that prophylactic dexamethasone is associated with a reduced risk of radiation-induced pain flare in patients with bone metastases receiving palliative radiotherapy. While these findings support a potential role for dexamethasone as an adjunctive strategy for symptom management, the current evidence base remains limited. Additional high-quality randomized controlled trials are required to confirm these findings, further evaluate safety outcomes, and establish evidence-based recommendations for clinical practice.

## Data Availability

The datasets presented in this study can be found in online repositories. The names of the repository/repositories and accession number(s) can be found in the article/supplementary material.
